# Change in quality of life and their predictors in the long-term follow-up after group cognitive behavioral therapy for social anxiety disorder: a prospective cohort study

**DOI:** 10.1186/1471-244X-10-81

**Published:** 2010-10-14

**Authors:** Norio Watanabe, Toshi A Furukawa, Junwen Chen, Yoshihiro Kinoshita, Yumi Nakano, Sei Ogawa, Tadashi Funayama, Tetsuji Ietsugu, Yumiko Noda

**Affiliations:** 1 Department of Psychiatry and Cognitive-Behavioral Medicine, Nagoya City University Graduate School of Medical Sciences, Nagoya, Japan; 2 Nagoya Keizai University Junior College, Inuyama, Aichi, Japan

## Abstract

**Background:**

Social anxiety disorder (SAD) is one of the most common anxiety disorders. The efficacy of cognitive behaviour therapy (CBT) has been examined but to date its effects on Quality of Life (QoL) have not been appropriately evaluated especially in the long term.

The study aimed to examine, in the long term, what aspects of Quality of Life (QoL) changed among social anxiety disorder (SAD) patients treated with group cognitive behaviour therapy (CBT) and what predictors at baseline were associated with QoL.

**Methods:**

Outpatients diagnosed with SAD were enrolled into group CBT, and assessed at follow-ups for up to 12 months in a typical clinical setting. QoL was evaluated using the Short Form 36. Various aspects of SAD symptomatology were also assessed. Each of the QoL domains and scores on symptomatology were quantified and compared with those at baseline. Baseline predictors of QoL outcomes at follow-up were investigated.

**Results:**

Fifty-seven outpatients were enrolled into group CBT for SAD, 48 completed the whole program, and 44 and 40 completed assessments at the 3-month and 12-month follow-ups, respectively. All aspects of SAD symptomatology and psychological subscales of the QoL showed statistically significant improvement throughout follow-ups for up to 12 months. In terms of social functioning, no statistically significant improvement was observed at either follow-up point except for post-treatment. No consistently significant pre-treatment predictors were observed.

**Conclusions:**

After group CBT, SAD symptomatology and some aspects of QoL improved and this improvement was maintained for up to 12 months, but the social functioning domain did not prove any significant change statistically. Considering the limited effects of CBT on QoL, especially for social functioning, more powerful treatments are needed.

## Background

Social anxiety disorder (SAD), also known as social phobia, is one of the most common psychiatric disorders, with a 12-month and lifetime prevalence of 7% [1] and 12% [2], respectively. SAD typically begins during the early teenage years and has a chronic course [2]. For example, prospective, long-term, naturalistic studies have indicated that only one-third of individuals attain remission from SAD within 8 years [3]. People with SAD are also at great risk for comorbid depression [4,5] and other anxiety disorders [6].

SAD is associated with significant disability and diminished quality of life (QoL) [7,8], which refers not only to one's subjective judgment of the satisfaction with everyday life, but also to objective indicators such as health status and external life situations [9]. Diagnostic-specific symptom measures for anxiety disorders explained only a small proportion of the variance in QoL [10,11], suggesting that an individual's perception of quality of life is an additional factor that should be part of a complete assessment. Depressive comorbidity in SAD contributes only modestly to the deterioration in QoL [8].

With regards to treatment for SAD, a large number of randomized controlled trials (RCTs) have investigated the efficacy of various types of pharmacotherapy and psychosocial intervention, and SAD is now regarded as a treatable condition [12]. According to meta-analyses, selective serotonin reuptake inhibitors (SSRIs) had a mean effect size between 1.3 and 1.9 in symptomatology scales in comparison with placebo [13], while cognitive behavioural therapy (CBT) encompassing exposure therapy and cognitive restructuring had a mean effect size of 0.8 in comparison with waiting list control [14].

QoL can also be improved with active treatment. In comparison with patients treated with placebo pills, several RCTs reported improvements in some QoL measures after treatment with a variety of antidepressants [15-17]. In terms of psychotherapy, improvements in some QoL measures have been reported in RCTs investigating the efficacy of CBT and subsequent social skills training [18], individual cognitive therapy [19], exposure therapy [20], internet-based CBT plus in vivo exposure [21], and internet-delivered CBT alone [22].

However, these studies have several limitations. First, studies on QoL in the longer term after psychosocial therapy are scarce, although SAD typically has a chronic course [2], and evaluations of treatment outcomes must consider the durability of gains after initial progress has been achieved. Second, QoL has often been reported by being aggregated into one [19,21,22] or two scales (mental health and physical health subscales) [20], but assessment of QoL has been reported that it should comprise at least the following four domains: physical functional status, disease and treatment-related physical symptoms, psychological functioning and social functioning [23]. Actually, a previous study [24] investigating QoL domains Short Form 36 [25] in college students reported those with social phobia were significantly associated with lower quality of life, particularly in general health, vitality, social functioning, role functioning-emotional, and mental health dimensions. Third, to date, predictors for better outcomes in QoL in the long-term after CBT have not been established, although several factors including sex and subtype of SAD were found to be associated with better outcomes in SAD symptomatology in one study [26].

We therefore aimed to examine: 1) what aspects of QoL change during long-term follow-up after group CBT in a typical clinical setting in a psychiatric clinic; 2) whether changes in the severity of symptomatology of SAD are directly associated with QoL at long-term follow-up; and 3) what predictors at baseline are associated with QoL in the long-term after group CBT.

We hypothesized that the improvement in QoL in the long-term after CBT would be: 1) shown in both psychological and social functioning domains; 2) associated with improvement in SAD symptomatology in the long term as well as in the short term; 3) and associated with low severity of SAD symptomatology, non-generalized SAD and good family support at baseline.

## Methods

### Patients

Details of the inclusion criteria for the participants and the contents of the group CBT as an acute-phase treatment have been described elsewhere [27]. In brief, 57 consecutive patients with SAD were initially recruited into the outpatient group-based CBT program at the Department of Psychiatry and Cognitive-Behavioral Medicine, Nagoya City University Hospital, Japan, between February 2005 and May 2007. Some of the patients were referred from mental health professionals and others sought treatment themselves.

All patients were diagnosed with DSM-IV SAD as the primary disorder using the Structured Clinical Interview for DSM-IV [28]. All patients also fulfilled the following criteria: (a) absence of a history of psychosis or bipolar disorder or of current substance use disorder; (b) no previous CBT treatments and no any other additional structured psychosocial therapies during the treatment period; and (c) absence of Cluster B personality disorders. Patients with current major depressive disorder, other current anxiety disorders and Cluster A and C personality disorders were included, when these symptoms abated sufficiently to allow them to attend the group CBT sessions regularly, judged by their physicians.

The patients provided their written informed consent after a full explanation of the objectives and procedures of the present study. The study protocol was approved by the Ethics Committee of the Nagoya City University Graduate School of Medical Sciences.

### Treatment

The CBT program consisted of 12 or more, two-hour, group sessions, with the number of sessions depending on each group's progress (maximum 20 sessions), and was based on Andrews et al's treatment manual [29]. The main components included psychoeducation, attention training, video-feedback of role-plays, behavioural experiments, cognitive restructuring and optional self-assertion training. Homework was actively tailored for each patient through collaboration of therapists and patients according to contents in each session, assigned after every session, and reviewed in subsequent sessions.

The patients were treated in groups of 3 or 4 led by two therapists (one principal and one co-therapist). Eight therapists (five psychiatrists and three doctoral-level clinical psychologists) each with more than three years of clinical practice and experience in treatment of anxiety disorders conducted the treatment program, guided by a therapists' manual. During the treatment, the therapists had group discussions once a month to check on therapist adherence to the program and to plan for future sessions.

During and after the CBT, co-administration of antidepressants and benzodiazepines was allowed as a part of usual treatment at a specialist clinic, because the present study was intended to reflect the outcomes of a typical clinical setting. No patient participated in other structured psychosocial treatments or other clinical research into SAD.

### Assessment

Demographic and diagnostic characteristics of the patients were gathered at baseline, including sociodemographic factors such as sex, age, education, marital status, living situation and employment status. Information about age of onset and duration of SAD, subtypes of SAD, psychiatric comorbidity (especially avoidant personality disorder) and medication use were also obtained.

The patients were assessed with an extensive questionnaire battery using observer-rated assessments and self-report questionnaires at baseline, post-treatment and at 3- and 12-month follow-ups. In addition to a questionnaire measuring various aspects of QoL, questionnaires on SAD symptomatology, including depression, were administered at each time point.

QoL was assessed using the Short Form 36 (SF-36) and severity of SAD was assessed using the Social Phobia Scale (SPS) and the Social Interaction Anxiety (SIAS). Depression was assessed as one aspect of SAD symptomatology using the Symptom Checklist-90-Revised (SCL-90-R).

#### Short Form 36 (SF-36)

The Japanese version of the Short Form 36 (SF-36 version 1.2) was used to assess QoL. The SF-36 [25] is a 36-item self-report questionnaire and is among the most frequently-used measures to evaluate health-related QoL. The SF-36 addresses both physical and emotional health states and provides validated scores indicating health variations in eight domains: physical functioning, role physical, bodily pain, general health perception, vitality, social functioning, role emotional and mental health. Each domain is scored from 0 to 100, with a higher score indicating better function. The SF-36 is thought to be able to address all necessary factors to measure QoL comprehensively [23]. The Japanese version had already been developed and validated [30].

#### Social Phobia Scale and Social Interaction Anxiety (SPS/SIAS)

The SPS and the SIAS [31] are 20-item self-report questionnaires. The SPS was designed to measure the fear of being observed, whereas the SIAS provides a measure of fear of social interaction. The items are rated on a 4-point scale from 0 (not at all characteristic or true of me) to 4 (extremely characteristic or true of me), with scores for each scale ranging from 0 to 80 and a higher score indicating a worse condition. Excellent internal consistency and reliability and sufficient predictive and concurrent validity have been demonstrated for both Japanese versions [32].

#### Symptom Checklist-90-Revised (SCL-90-R)

The SCL-90-R is a 90-item questionnaire widely used to assess general psychopathology [33]. A higher score indicates worse status for each dimension. The reliability and validity of the Japanese version have been demonstrated [34]. We used the depression subscale of this comprehensive psychology scale.

### Statistical analysis

All patients who completed the group CBT and whose data were obtained at the follow-ups were included in the analyses. All analyses were conducted as completer analyses, where data from patients who completed the post-treatment and follow-up assessments were considered. An intention-to-treat analysis, where data from all patients who were enrolled into the study were considered, was not conducted, but we performed one-way ANOVA for continuous variables or χ^2 ^tests for categorical variables to compare QoL, demographic data and SAD symptomatology between completers and dropouts from the program or follow-up assessments.

All the statistical tests were two-tailed, and an alpha value of less than 0.05 was considered statistically significant. Results with an alpha value of less than 0.005 were also identified, since multiple tests were conducted in the analysis and we did not use any formal methods to correct this, given that the study is the first detailed, systematic evaluation of QoL domains in the long term and was therefore considered to be an exploratory study.

All the data were analyzed using SPSS 16.0 for Windows [35].

#### Changes in symptoms and QoL through the treatment and follow-ups

The outcomes of the CBT program for the patients with SAD were qualified using paired t-tests between pre-treatment and each follow-up time point (post-treatment and 3- and 12-month follow-ups) in terms of the QoL scores (eight domains of the SF-36) as well as the SAD symptomatology scores (SPS, SIAS and SCL-90-R depression status). The magnitude of any differences was calculated as an effect size [(mean follow-up - mean pre-treatment)/pooled SD] with 95% confidence intervals. Effect sizes are usually categorized as follows: small (0.20-0.49), medium (0.50-0.79), and large effects (0.80 and above) [36].

#### Correlation between changes in SAD symptomatology and those in QoL

Tests of correlation were undertaken and Pearson's *r *was calculated with the differences between pre-treatment and each follow-up time point (pot-treatment and 3- and 12-month follow-ups) on the eight domain-scores of the SF-36 and the symptomatological measures of SAD.

#### Potential predictors at baseline for changes in QoL at follow-ups

In order to elucidate the baseline predictors of the treatment outcomes at post-treatment and the 3- and 12-month follow-ups, multiple regression analyses using a stepwise method (probability of F to enter, ≤ 0.50; to remove, ≥0.10) were conducted with the eight domain scores of the SF-36 at each time point as dependent variables and the baseline demographic and clinical variables as independent variables, controlling for the baseline SF-36 score.

## Results

### Demographic and clinical characteristics of the patients

Fifty-seven outpatients were initially enrolled into group CBT (Table 1). No patients satisfied the diagnostic criteria of avoidant personality disorder according to DSM-IV. Of these enrolled into the CBT program, 48 completed the program, and 44 and 40 completed the assessments at the 3-month and 12-month follow-ups, respectively. The demographic characteristics, SAD symptomatology and QoL at baseline did not significantly differ among patients who dropped out during the CBT program or follow-up prematurely, apart from living situation (Table 1).

**Table 1:** Demographic and clinical characteristics of the patients at baseline

	Patients who entered the CBT but did not complete	Patients who completed the CBT but not the 3-month FU assessments	Patients who completed the the 3-month but not the 12-month FU assessments	Patients who provided both FU assessments	P value
**Number of subjects**	**9**	**4**	**4**	**40**	

**Female, No. (%)**	3 (33.3)	2 (50.0)	2 (50.0)	23 (57.5)	0.63
**Age, years**					
**Mean ± SD**	27.4 ± 8.8	34.2 ± 9.3	34.2 ± 9.3	34.2 ± 8.9	0.12
**Range**	18-47	18-52	18-52	18-52	
**Onset of SAD, years**					
**Mean ± SD**	17.1 ± 4.2	19.4 ± 7.8	19.4 ± 7.8	19.6 ± 7.8	0.12
**Range**	12-23	5-42	5-42	5-42	
**Living situation, No (%)**					0.03*
**With spouse/significant-other**	0 (0.0)	1 (25.0)	2 (50.0)	20 (50.0)	
**With parents**	5 (55.6)	3 (75.0)	2 (50.0)	16 (40.0)	
**Alone**	4 (44.4)	0 (0.0)	0 (0.0)	4 (10.0)	
**Employment**					0.61
**Full-time employment**	2 (22.2)	1 (25.0)	1 (25.0)	10 (25.0)	
**Full-time student**	2 (22.2)	0 (0.0)	0 (0.0)	2 (5.0)	
**Part-time/homemaker/retired**	3 (33.3)	2 (50.0)	3 (75.0)	23 (55.0)	
**Unemployed**	2 (22.2)	1 (25.0)	0 (0.0)	6 (15.0)	
**Education, No. (%)**					0.61
**University**	2 (22.2)	1 (25.0)	1 (25.0)	15 (37.5)	
**College**	1 (11.1)	3 (75.0)	1 (25.0)	10 (25.0)	
**High school or less**	6 (66.6)	0 (0.0)	2 (50.0)	15 (37.5)	
**Social phobia, No. (%) generalized**	8 (88.8)	4 (100.0)	3 (75.0)	34 (85.0)	0.72
**Comorbidity, No. (%)**					
**Mood disorders**	2 (22.2)	4 (100.0)	2 (50.0)	14 (35.0)	0.05
**Anxiety disorders**	1 (11.1)	1 (25.0)	0 (0.0)	4 (10.0)	0.71
**Medication, No. (%)**					
**Benzodiazepine**	4 (44.4)	0 (0.0)	1 (25.0)	11 (27.5)	0.20
**Antidepressant**	7 (77.8)	2 (50.0)	2 (50.0)	20 (50.0)	0.50
**SF-36**					
**Physical functioning**	85.6 ± 14.7	91.3 ± 11.8	82.5 ± 22.2	88.6 ± 14.3	0.79
**Role-physical**	75.0 ± 43.3	62.5 ± 43.3	56.3 ± 51.5	68.6 ± 36.6	0.87
**Bodily pain**	90.3 ± 19.4	64.3 ± 29.4	66.5 ± 32.5	65.8 ± 25.3	0.08
**General health perception**	47.2 ± 22.4	49.8 ± 26.3	44.3 ± 26.3	45.1 ± 22.1	0.98
**Vitality**	42.2 ± 23.6	45.0 ± 5.8	20.0 ± 18.3	39.6 ± 21.1	0.28
**Social functioning**	45.8 ± 23.4	56.3 ± 21.7	65.6 ± 38.7	58.7 ± 30.2	0.63
**Role-emotional**	51.9 ± 44.4	50.0 ± 43.0	41.7 ± 50.0	57.3 ± 40.4	0.89
**Mental health**	44.9 ± 14.9	48.0 ± 3.3	36.0 ± 15.0	47.9 ± 20.5	0.67
**SPS (total)**	44.0 ± 13.7	47.3 ± 28.0	34.0 ± 6.2	33.7 ± 12.5	0.08
**SIAS (total)**	55.3 ± 9.8	64.5 ± 26.4	51.3 ± 11.2	51.0 ± 13.3	0.29
**SCL-90-R depression**	1.67 ± 0.96	1.52 ± 0.75	1.35 ± 0.89	1.50 ± 0.87	0.93

P-values were calculated using one-way ANOVA for continuous variables and using χ^2 ^statistic for categorical variables.

**Appendices: **FU, follow-up; SAD, social anxiety disorder; SCL-90-R, Symptom Checklist-90-Revised; SF-36, Short Form 36; SIAS, Social Interaction Anxiety; SPS, Social Phobia Scale

(* P < 0.05, ** P < 0.005)

### Changes in symptoms and QoL through the treatment and follow-ups

Examination of changes in all the SAD symptomatology measures between pre-treatment and each subsequent time point revealed significant improvement not only at post-treatment but also up to 12-month follow-up, with an effect size of -0.96 (large effect) on SPS, of -0.87 (large effect) on SIAS and of -0.45 (small effect) on SCL-90-R depression at 12-month follow-up (Table 2). In terms of the QoL domains, general health perception, vitality and mental health were statistically significantly better post-treatment and these improvements were maintained for up to 12 months of follow-up, with an effect size of 0.44 (small effect), of 0.29 (small effect), and of 0.32 (small effect) at 12-month follow-up, respectively. However, in terms of social functioning, no statistically significant improvement was observed at follow-up apart from at post-treatment with an effect size of 0.30 (small effect).

**Table 2:** Mean symptom scores at follow-ups and effect sizes in comparison with those at baseline

		Pre-treatment	Post-treatment	3-month FU	12-month FU
**Number of the patients**		**57**	**48**	**44**	**40**

**SF-36**					
**Physical functioning**	**Score**	87.9 (14.5)	89.8 (14.5)	89.3 (15.4)	90.6 (14.8)
	**ES**		0.10 (-0.32, 0.51)	0.02 (-0.41, 0.46)	0.15 (-0.30, 0.59)
	
**Role-physical**	**Score**	68.3 (38.3)	72.8 (34.5)	78.0 (35.0)	81.9 (31.5)*
	**ES**		0.06 (-0.36, 0.48)	0.27 (-0.17, 0.70)	0.39 (-0.06, 0.84)
	
**Bodily pain**	**Score**	69.7 (26.1)	70.8 (21.8)	75.1 (23.9)*	67.3 (24.6)
	**ES**		0.24 (-0.19, 0.65)	0.32 (-0.12, 0.75)	0.12 (-0.32, 0.57)
	
**General health perception**	**Score**	45.7 (22.1)	53.2 (22.3)*	54.3 (24.7)*	55.1 (24.4)*
	**ES**		0.28 (-0.14, 0.70)	0.35 (-0.09, 0.78)	0.44 (-0.02, 0.89)
	
**Vitality**	**Score**	39.0 (21.0)	49.2 (17.8)**	46.5 (24.6)**	45.3 (22.4)*
	**ES**		0.51 (0.08, 0.93)	0.36 (-0.08, 0.79)	0.29 (-0.17, 0.74)
	
**Social functioning**	**Score**	56.9 (29.1)	68.6 (29.6)*	67.3 (28.9)	66.3 (26.6)
	**ES**		0.30 (-0.12, 0.72)	0.26 (-0.17, 0.70)	0.28 (-0.17, 0.72)
	
**Role-emotional**	**Score**	54.8 (40.9)	61.5 (43.8)	67.5 (38.4)	60.8 (43.3)
	**ES**		0.07 (-0.35, 0.49)	0.31 (-0.13, 0.75)	0.08 (-0.36, 0.52)
	
**Mental health**	**Score**	46.6 (18.6)	54.4 (16.6)*	55.4 (21.3)*	54.7 (23.9)*
	**ES**		0.39 (-0.04, 0.81)	0.40 (-0.04, 0.83)	0.32 (-0.13, 0.77)

**SPS (total)**	**Score**	36.3 (14.2)	24.9 (14.8)**	20.4 (11.0)**	21.8 (12.7)**
	**ES**		-0.69 (-1.10, -0.28)	-1.14 (-1.59, -0.68)	-0.96 (-1.41, -0.50)

**SIAS (total)**	**Score**	52.7 (13.9)	41.4 (15.6)**	37.8 (13.1)**	38.4 (15.5)**
	**ES**		-0.71 (-1.12, -0.29)	-1.00 (-1.44, -0.54)	-0.87 (-1.32, -0.41)

**SCL-90-R depression**	**Score**	1.52 (0.86)	0.96 (0.75)**	1.08 (0.86)**	1.09 (0.97)**
	**ES**		-0.66 (-1.07, -0.24)	-0.44 (-0.86, -0.01)	-0.45 (-0.89, 0.00)

Scores are presented with SDs (in parentheses), and ESs with their 95% confidence intervals. ES calculations and paired *t *tests were conducted for completers at each time point, by comparing scores with those at baseline.

**Appendices: **ES, effect size; FU, follow-up; SCL-90-R, Symptom Checklist-90-Revised; SF-36, Short Form 36; SIAS, Social Interaction Anxiety; SPS, Social Phobia Scale

(* P < 0.05, ** P < 0.005)

### Correlation between changes in SAD symptomatology and those in QoL

Changes in the total scores of each of the SAD symptomatology measures significantly correlated with changes in the mental health domain score throughout the follow-up period, with a Pearson's *r *of around -0.5 (Table 3). Change in the SIAS score was significantly associated with change in the role emotional domain through the entire period of follow-up, with a Pearson's *r *of around -0.35, whilst those in the SPS score and the depression score were associated with that of role emotional only at post-treatment. In contrast, the change in the SPS score was significantly associated with the change in the social functioning domain not at post-treatment but at the 3- and 12-month follow-ups, with a Pearson's *r *of around -0.4, whilst the changes in the SIAS score and depression score were associated with changes in social functioning only at post-treatment.

**Table 3:** Correlation between changes in SAD symptomatology and those in QoL between pre-treatment and follow-ups

	Post-treatment			3-month follow-up			12-month follow-up		
**Number of the patients**	**48**			**44**			**40**		

	**SPS**	**SIAS**	**SCL-90-R Depression**	**SPS**	**SIAS**	**SCL-90-R Depression**	**SPS**	**SIAS**	**SCL-90-R Depression**

**SF-36**									
**Physical functioning**	-0.32*	-0.19	-0.33*	-0.23	-0.18	-0.27	-0.09	-0.03	-0.05
**Role-physical**	-0.13	-0.16	-0.12	-0.31*	-0.22	-0.33*	-0.12	-0.02	-0.10
**Bodily pain**	-0.30	-0.17	-0.05	-0.51**	-0.46**	-0.31	-0.11	-0.19	0.07
**General health perception**	-0.43**	-0.41*	-0.33*	-0.53**	-0.47**	-0.47**	-0.28	-0.26	-0.31
**Vitality**	-0.16	-0.34*	-0.44**	-0.56**	-0.64**	-0.36*	-0.43*	-0.58**	-0.36*
**Social functioning**	-0.22	-0.35*	-0.41*	-0.37*	-0.27	-0.48**	-0.44*	-0.17	-0.26
**Role-emotional**	-0.45**	-0.36*	-0.41*	-0.28	-0.36*	-0.14	-0.24	-0.33*	-0.22
**Mental health**	-0.42*	-0.53**	-0.53**	-0.52**	-0.62**	-0.50**	-0.44*	-0.54**	-0.45*

Pearson's *r*s are presented in the table.

**Appendices: **SCL-90-R, Symptom Checklist-90-Revised; SF-36, Short Form 36; SIAS, Social Interaction Anxiety; SPS, Social Phobia Scale

(* P < 0.05, ** P < 0.005)

### Potential predictors at baseline for changes in QoL at follow-ups

None of the symptomatology scores for SAD at baseline were significant predictors of social functioning post-treatment or at 3 months, but all were significant predictors at 12 months (Table 4). Depression at baseline was significantly associated with the role emotional domain throughout follow-up, apart from at post-treatment. No significant pre-treatment predictors throughout the entire period of follow-up were identified for any of the QoL outcomes.

**Table 4:** Predictors at baseline for changes at follow-ups in QoL in SAD patients treated with CBT

Characteristics	Post treatment								3-month follow-up								12-month follow-up							
**at baseline**	**PF**	**RP**	**BP**	**GH**	**VT**	**SF**	**RE**	**MH**	**PF**	**RP**	**BP**	**GH**	**VT**	**SF**	**RE**	**MH**	**PF**	**RP**	**BP**	**GH**	**VT**	**SF**	**RE**	**MH**

**Adjusted R-square**	0.62	0.12	0.17	0.39	0.50	0.27	0.15	0.41	0.45	0.25	0.23	0.47	0.59	0.36	0.19	0.32	0.64	0.21	0.33	0.29	0.46	0.55	0.35	0.37

**Age: 35 yrs or older**	a	a	a	a	a	a	0.41*	a	a	a	a	a	a	a	a	a	a	a	a	a	a	a	a	a
**Living situation**	a	a	a	a	-0.32**	a	a	a	a	a	a	a	a	a	a	a	a	a	a	a	a	a	a	a
**Employment**	a	a	a	a	a	a	a	a	a	a	a	a	-0.30*	a	a	a	a	a	a	a	a	a	a	a
**Generalized SAD**	-0.22*	a	a	a	a	a	a	-0.28*	a	a	a	a	a	a	a	a	a	a	a	a	a	a	a	a
**Benzodiazepine use**	0.23*	a	a	a	a	a	a	a	a	a	a	a	a	a	a	a	a	a	a	a	a	a	a	a
**SPS (total)**	a	a	a	a	a	a	a	a	a	0.39*	a	0.29*	a	a	a	a	a	a	a	a	a	0.49**	a	a
**SIAS (total)**	a	a	a	a	a	a	a	a	a	a	a	a	a	a	a	a	a	a	a	a	a	-0.32*	a	a
**SCL-90-R depression**	a	a	-0.44**	a	a	a	a	a	a	-0.64**	a	a	a	a	-0.46**	a	a	a	a	a	a	-0.41*	-0.61**	a

Table shows the standardized Beta coefficients, except for a row showing adjusted R-squares of the models. Sex, age of onset (20 years or older), marital status, education, comorbid mood or anxiety disorder, and antidepressant use were entered but not selected in any regression models through application of a stepwise method.

^a ^Entered into the analysis but not selected in the multiple regression model through application of a stepwise method.

**Appendices: **PF, physical functioning; RP, role physical; BP, bodily pain; GH, general health perception; VT, vitality; SF, social functioning; RE, role emotional; MH, mental health; SAD, social anxiety disorder; SCL-90-R, Symptom Checklist-90-Revised; SF-36, Short Form 36; SIAS, Social Interaction Anxiety; SPS, Social Phobia Scale

(* P < 0.05, ** P < 0.005)

## Discussion

### Main findings

To our knowledge, this is the first detailed evaluation of long-term QoL after a CBT program for SAD. We assessed patients for up to 12 months after the cessation of group CBT provided in a typical clinical setting. The study revealed several important findings.

First, SAD symptomatology and some aspects of QoL did improve and these improvements were maintained for at least 12 months after group CBT. However, the improvement in the social functioning domain of the SF-36, which was noted post-treatment, was not maintained over the 12 months of follow-up.

Second, social functioning at follow-up through to 12 months was not always associated with improvements in SAD symptomatology, especially the SIAS. A previous study of group CBT concluded that QoL in one aggregated scale post-treatment was associated with the SIAS among patients diagnosed with social phobia [37].

Third, we hypothesized that a low severity of SAD symptomatology, non-generalized SAD, and good family support would be associated with better outcomes in QoL, as suggested by previous research [26,37,38], but no consistent pre-treatment predictors were detected for any of the QoL domains throughout follow-up for up to 12 months.

The most striking finding of the current study may be that the social functioning domain, which is significantly impaired in patients with SAD in comparison with the general population [39], improved post-treatment, but the degree of improvement was very small (effect size on social functioning, 0.30) and was not maintained at follow-up, although scores of SAD symptomatology were much improved (effect size, around 1.0). Attention should be paid to this discrepancy between QoL and SAD symptomatology, because patients may judge the outcome of therapy based on their subjective feelings of QoL while clinicians may judge outcome based on diagnostic and symptom measures [40]. A previous report concluded that group CBT led to significant improvement in the social functioning factor of a QoL scale in SAD patients [41], but the magnitude of this improvement was not reported. The effect size calculated by using pre- and post-treatment scores and pre-test standard deviations of the social functioning factor in the report was 0.40, which is similar to the value of the effect size in our study, so we should be cautious about concluding that CBT offers promising improvements in social functioning.

Considering the small and short-term effects of CBT on QoL, especially for social functioning, more powerful treatments are needed in clinical practice. Although a previous study has reported that cognitive therapy with no formal social skills training led to significantly more improvement in SAD symptomatology than exposure plus applied relaxation or wait-list did[42], no QoL outcomes were reported in this study. Other treatment factors, such as social skills training, might have an additional benefit to social functioning in the long term. Future studies should place more focus on social functioning rather than on SAD symptomatology.

### Limitations

The present study is not without its methodological limitations.

First, the study was conducted as a single-arm, naturalistic, follow-up study and was no control condition was used. An RCT with an appropriate control group is therefore needed to investigate the efficacy of treatment. Moreover, any antidepressant and benzodiazepine medications were allowed at baseline. The information about changes in dosing were not collected. Medications might have had an effect on the outcomes and this issue should be listed among limitations, although most of the patients had suffered from social anxiety disorders for more than 10 years and had already been on medication for a long time. However, this study was intended to examine the long-term consequences of CBT through naturalistic follow-up in a typical clinical setting. We believe that the study design is appropriate for this purpose.

Second, the sample size of the study might have been too small to identify statistically significant changes in the domains of the SF-36 or to detect potential baseline predictors of the SF-36 at the follow-ups. Nevertheless, the changes of each of the SAD symptomatology scores were statistically significant, with an effect size of around 1.0. The effect size of social functioning, which we hypothesized would improve significantly, reached a maximum of only 0.3 during follow-up, which must be considered a small effect, if indeed there is any effect at all.

Third, one may argue that, instead of the SPS and SIAS, a more frequently-used measure such as the Liebowitz Social Anxiety Scale (LSAS) should have been used to evaluate SAD symptomatology. The LSAS is a 24-item scale that provides separate scores for fear and avoidance of social interaction and performance situations, and the Japanese version has sufficient validity data [43]. We did not use it because assessments at the follow-ups were done by patient self-evaluation and the LSAS requires an assessor. Although one paper reported data supporting the use of the LSAS as a self-reporting instrument [44], we were not willing to use the LSAS in an unconventional way because a self-reporting version has not yet been validated in Japan.

Fourth, a standard treatment manual [29] has been adopted, we did not conduct any booster sessions after the acute-phase treatment. This might have effect on the fact that the improvement at post-treatment in the social functioning domain in the SF-36 were not maintained over a 12-month follow-up period, although that in SAD symptomatological outcomes were maintained. Future studies might be needed to investigate the efficacy of booster sessions on social functioning outcomes.

## Conclusions

Symptomatology of SAD and some aspects of QoL improved, and these improvements were maintained for up to 12 months, after group CBT but the social functioning domain did not significantly change. Better treatments for SAD, focusing on improving social functioning, are needed in clinical practice.

## Competing interests

The authors declare that they have no competing interests.

## Authors' contributions

NW conceived of the study, performed the clinical investigation (diagnosis, treatment and assessment), and drafted the manuscript. TAF participated in the design of the study and performed the clinical investigation. JC, YNa, YK, SO, TF, TI, and YNo performed the clinical investigation. All authors read and approved the final manuscript.

## Pre-publication history

The pre-publication history for this paper can be accessed here:

http://www.biomedcentral.com/1471-244X/10/81/prepub
